# Celiac disease biomarkers identified by transcriptome analysis of small intestinal biopsies

**DOI:** 10.1007/s00018-018-2898-5

**Published:** 2018-08-10

**Authors:** Hanna Bragde, Ulf Jansson, Mats Fredrikson, Ewa Grodzinsky, Jan Söderman

**Affiliations:** 1 Laboratory Medicine, Ryhov County Hospital, Building E3 Level 4, 55185 Jönköping, Sweden; 2 Department of Clinical and Experimental Medicine, Faculty of Medicine and Health Sciences, Linköping University, Linköping, Sweden; 3 Department of Pediatrics, Ryhov County Hospital, Jönköping, Sweden; 4 Department of Clinical and Experimental Medicine and Forum Östergötland, Faculty of Medicine and Health Sciences, Linköping University, Linköping, Sweden; 5 Division of Forensic Genetics & Forensic Toxicology, National Board of Forensic Medicine, Linköping, Sweden; 6 Department of Medicine and Health, Faculty of Medicine and Health Sciences, Linköping University, Linköping, Sweden

**Keywords:** RNA-seq, RNA sequencing, Molecular biomarkers, Gene expression profiling, Gene ontology enrichment analysis

## Abstract

Establishing a celiac disease (CD) diagnosis can be difficult, such as when CD-specific antibody levels are just above cutoff or when small intestinal biopsies show low-grade injuries. To investigate the biological pathways involved in CD and select potential biomarkers to aid in CD diagnosis, RNA sequencing of duodenal biopsies from subjects with either confirmed Active CD (*n* = 20) or without any signs of CD (*n* = 20) was performed. Gene enrichment and pathway analysis highlighted contexts, such as immune response, microbial infection, phagocytosis, intestinal barrier function, metabolism, and transportation. Twenty-nine potential CD biomarkers were selected based on differential expression and biological context. The biomarkers were validated by real-time polymerase chain reaction of eight RNA sequencing study subjects, and further investigated using an independent study group (*n* = 43) consisting of subjects not affected by CD, with a clear diagnosis of CD on either a gluten-containing or a gluten-free diet, or with low-grade intestinal injury. Selected biomarkers were able to classify subjects with clear CD/non-CD status, and a subset of the biomarkers (*CXCL10*, *GBP5*, *IFI27*, *IFNG*, and *UBD*) showed differential expression in biopsies from subjects with no or low-grade intestinal injury that received a CD diagnosis based on biopsies taken at a later time point. A large number of pathways are involved in CD pathogenesis, and gene expression is affected in CD mucosa already in low-grade intestinal injuries. RNA sequencing of low-grade intestinal injuries might discover pathways and biomarkers involved in early stages of CD pathogenesis.

**Electronic supplementary material:**

The online version of this article (10.1007/s00018-018-2898-5) contains supplementary material, which is available to authorized users.

## Introduction

Celiac disease (CD) is an immune-mediated systemic disorder elicited by gluten and related prolamines in genetically susceptible individuals, with the presence of a variable combination of gluten-dependent clinical manifestations, CD-specific antibodies, such as IgA autoantibodies against tissue transglutaminase (anti-TG2), human leukocyte antigen (HLA)-DQ2 or HLA-DQ8 haplotypes, and enteropathy [1]. The histological alterations in the small intestine can be graded according to the modified Marsh scale [2, 3], and age has been shown to correlate inversely with intestinal lesion severity and anti-TG2 levels [4]. However, for children under 2 years of age, anti-TG2 levels can be below cutoff despite presence of Marsh grade 3 intestinal lesions [5]. The presence of anti-TG2 in the blood combined with Marsh grade 3 intestinal lesions is a strong indicator of CD, but diagnosis is less clear in cases with histopathology of Marsh grade 1–2 or in cases with only slightly elevated anti-TG2 levels [6]. The high prevalence of selective IgA deficiency in CD patients further complicates diagnosis [7]. Additionally, the determination of CD diagnoses may be difficult due to patchy distributions of lesions [8] or suboptimal orientations of small intestinal biopsies prepared for histopathologic assessment [9]. Furthermore, the amount of gluten consumed by an individual with CD and the amount of gluten that they can tolerate [10] affect enteropathy and CD-specific antibody levels.

It has been shown that the presence of HLA-DQ2 or HLA-DQ8 is essential, but not sufficient, for the development of CD [11], and genetic investigations of CD have identified 42 CD-associated non-HLA loci [12]. When RNA from specific intestinal compartments (surface epithelium, lamina propria, and crypts of Lieberkühn) [13] and RNA from whole intestinal biopsies [14–17] were evaluated, gene expression in the small intestines of CD study subjects with active disease differed from gene expression in the small intestines of study subjects without CD. Several CD gene expression studies have investigated the biological pathways required for the development and maintenance of enteropathy in CD using small intestinal biopsies [14–16], specific cell types [18, 19], and genetic approaches [20, 21]. However, we did not find any studies that evaluated whole intestinal biopsies by RNA sequencing; therefore, we sequenced RNA from small intestinal biopsies from study subjects without a CD diagnosis and study subjects with Active CD (Marsh grade 3) to conduct an unbiased investigation of differentially expressed genes (DEGs) and biological pathways in CD to improve CD diagnostics, especially for ambiguous cases, and to gain a better understanding of CD. We identified potential biomarkers for CD and validated them by real-time polymerase chain reaction (PCR) using study subjects with convincing Marsh grade 0 or Marsh grade 3 histologies and study subjects with low-grade intestinal injury.

## Materials and methods

### Study subjects

Pediatric patients in this study were referred to Ryhov County Hospital in Jönköping, Sweden, with suspected CD, or were followed-up after a period on a gluten-free diet (GFD) to verify mucosal recovery. Most patients were referred for small intestinal biopsy due to elevated anti-TG2 with or without symptoms. Patients with negative anti-TG2 (< 7 U/mL) with or without selective IgA deficiency on a gluten-containing diet (GD) were selected for small intestinal biopsy based on a clinical need to exclude CD (e.g., symptoms, hereditary factors, etc.). The patients were included in this study after written consent was provided, and blood and duodenal biopsy specimens were collected from all patients. This study was approved by the Regional Ethical Review Board in Linköping (2011/239-31).

### Study groups for RNA sequencing and validation of biomarkers

**Fig. 1:**
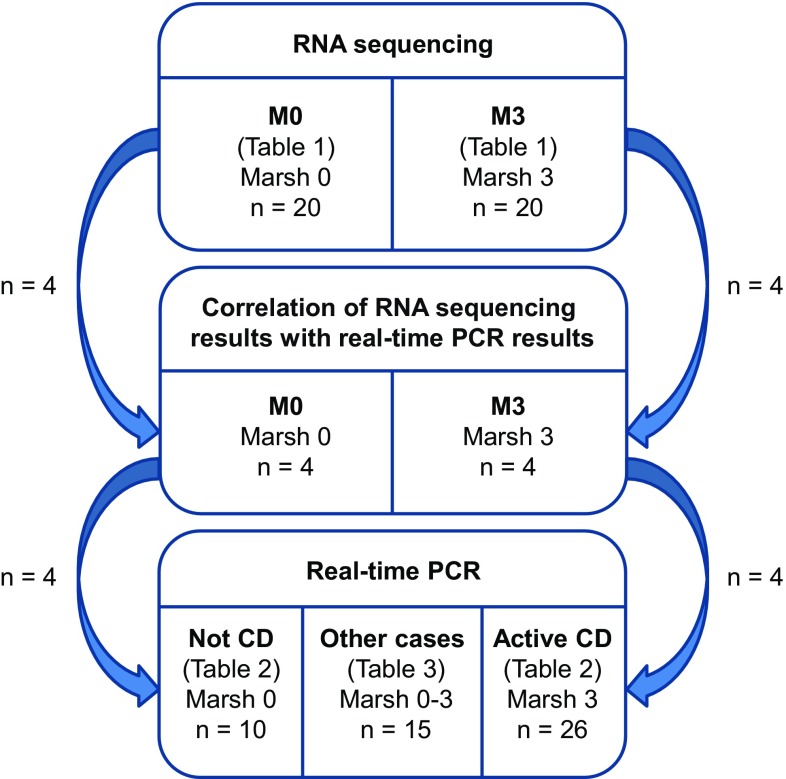
Flow diagram illustrating the number and type of study subjects included in the different parts of this study. RNA sequencing (upper section) was performed on 20 subjects without CD (study group M0) and 20 subjects with active CD of grade Marsh 3 (study group M3), which are described further in Table 1. Eight study subjects were selected from the RNA sequencing part and used for correlation between results from RNA sequencing and real-time PCR (midsection). Biopsies from these eight study subjects together with biopsies from 43 independent study subjects represent the entire set of 51 biopsies used for the follow-up study of potential CD biomarkers by means of real-time PCR (lower section). Additional data on these 51 study subjects can be found in Tables 2 and 3

Study subjects with a Marsh grade 0 histopathologic assessment and anti-TG2 < 7 U/mL were included in the RNA sequencing study group M0 and those with a Marsh grade 3 histopathologic assessment and anti-TG2 ≥ 7 U/mL were included in study group M3 (Table 1, Fig. 1). All of the study subjects were on a GD, and subjects in study group M3 received a CD diagnosis, whereas subjects in study group M0 did not. Two subjects in study group M0 had an IgA deficiency, but results from analysis of IgG antibody levels were available for TG2 and deamidated gliadin (DG).

**Table 1:** Descriptive statistics of the RNA sequencing study groups

Study group	*n*	Age at biopsy (years)^a^	Gender; M/F	Anti-TG2^a,b^ (U/mL)	Anti-DG^a,c^ (U/mL)	HLA-DQ2.5*cis*^d^
M0	20	8.5 (1.6–17)	10/10	0.20 (0–3.6)	0.50 (0–3.2)	0.65, 0.30, 0.050
M3	20	10 (2.3–18)	10/10	262 (36–2858)	89 (9–781)	0.15, 0.75, 0.10

Study group M0 contained study subjects with histopathologic assessments corresponding to grade Marsh 0, whereas group M3 contained study subjects with assessments corresponding to grades Marsh 3A, 3B, or 3C. All of the study subjects were on a gluten-containing diet, and subjects in study group M3 received a celiac disease diagnosis, whereas subjects in study group M0 did not

^a^Mean (min–max)

^b^Levels of IgA autoantibodies against tissue transglutaminase (anti-TG2) in serum. For two subjects in study group M0, no serum results were available, but plasma results were within the range of the serum results. IgG results from two subjects with IgA deficiency were included, which were within the range of the IgA-based results

^c^Levels of IgG antibodies against deamidated gliadin (anti-DG) in serum. For four subjects in study group M0 and one subject in study group M3, no serum results were available, but plasma results were within the range of the serum results

^d^For each group, the fractions of study subjects with 0, 1, or 2 HLA-DQ2.5*cis* are accounted for

The RNA sequencing results were validated by measuring gene expression levels of the selected potential CD biomarkers in four M0 and four M3 study subjects using real-time PCR (Fig. 1). For a validated result, we expected a high correlation between the two methods [product-moment correlation coefficient (*r*) ≥ 0.9], and the power to identify a correlation of this size at an α-level of 0.05 using a total of eight samples was 0.94 (G*Power version 3.1.9.2 [22]).

To further verify the differential expressions found by RNA sequencing, gene expressions of the potential CD biomarkers were analyzed in an independent set of study subjects with the same characteristics as the study subjects in the M0 and M3 groups [Not CD (*n* = 6) and Active CD (*n* = 22)] and in the previous eight study subjects [Not CD (*n* = 4) and Active CD (*n* = 4)] from validation of the RNA sequencing results (Table 2, Fig. 1). Using the Mann–Whitney *U* test at a Bonferroni-adjusted α-level of 0.0019 and these group sizes, the calculated power was 0.96 to detect a fold change (FC) > 4 or FC < − 4 (standard deviation = 2), which we used as the FC cutoff to select potential CD biomarkers. Study subjects in the Active CD group were selected to represent Marsh grade 3A (*n* = 8), 3B (*n* = 8), and 3C (*n* = 10) histopathologic assessments to investigate whether gene expressions correlated with Marsh grade. Results from gene expression analyses of the Not CD and Active CD study groups were used as a baseline for an additional analysis of fifteen study subjects with normalized mucosa on a GFD (study subjects 8–12, Table 3), Marsh 3 histopathology but negative anti-TG2 (study subjects 13–15, Table 3), or no or low-grade intestinal injury (study subjects 1–7, Table 3) (Fig. 1).

**Table 2:** Descriptive statistics of the two clear groups of study subjects used for the validation of RNA sequencing results by real-time polymerase chain reaction

Study group	*n* (M/F)	Marsh grade	Age (years)^a^	Diagnosis	Diet	Anti-TG2 (U/mL)^b^	HLA-DQ2.5*cis*^c^
Not CD	10 (2/8)	0	7.9 (1.1–17)	Not CD	GD	0.63 (0–3.6)	0.6, 0.4, 0
Active CD	26 (12/14)	3A–3C	7.8 (1.8–18)	CD	GD	712 (15–6832)	0.23, 0.62, 0.08

**Fig. 2:**
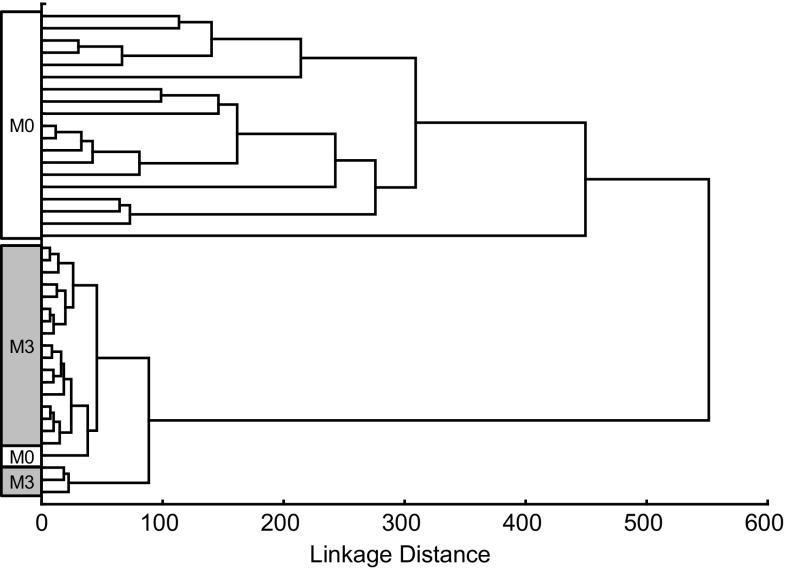
Hierarchical clustering of study subjects with histopathologic assessments corresponding to grade Marsh 3 (M3) or Marsh 0 (M0) based on RNA sequencing data (this study) from eight genes (*APOC3*, *CYP3A4*, *OCLN*, *MAD2L1*, *MKI67*, *CXCL11*, *IL17A*, and *CTLA4*) that were included in a previously developed gene expression profile

The Active CD group included study subjects with histopathologic assessments corresponding to grade Marsh 3 and elevated levels of IgA autoantibodies against tissue transglutaminase (anti-TG2) on a gluten-containing diet (GD). The Not CD group contained study subjects with histopathologic assessments corresponding to grade Marsh 0 and anti-TG2 levels below cutoff on a GD. The principal component analysis (Fig. 2) was constructed based on gene expressions from these two groups

^a^Age at biopsy, expressed as the mean (min–max)

^b^Levels of anti-TG2 analyzed in serum, expressed as the mean (min–max)

^c^For each group, the fractions of study subjects with 0, 1, or 2 HLA-DQ2.5*cis* are accounted for. Data was not available for two study subjects in group Active CD

**Table 3:** Descriptive statistics of study subjects used for the validation of RNA sequencing results by real-time polymerase chain reaction

Study subject (gender)	Marsh grade	Age (years)	Diagnosis	Context	Diet	Anti-TG2 (U/mL)^a^	HLA-DQ2.5*cis*^b^
1 (F)	0–2	3.1	CD	CD later^c^	GD	106	1
2 (M)	0	7.3	CD	CD later^c^	GD	70	1
3 (F)	1	15	CD	CD later^c^	GD	93	1
4 (F)	0	15	CD	CD later^c^	GD	10	1
5 (F)	0–1	9.1	Not CD	Not CD^d^	GD	23	1
6 (F)	2–3B	14	CD	CD	GD	27	0
7 (F)	2	16	CD	CD	GD	50	1
8 (M)	0	7	CD	Normalized CD	GFD	0.4	1
9 (F)	0	17	CD	Normalized CD	GFD	1.6	0
10 (F)	0	9	CD	Normalized CD	GFD	2.2	1
11 (F)	0	17	CD	Normalized CD	GFD	1.3	1
12 (F)	0	5	CD	Normalized CD	GFD	0.9	1
13 (F)	3C	0.7	CD	M3 TG-	GD	2.4	1
14 (F)	3C	0.8	CD	M3 TG-	GD	2.8	N/A
15 (F)	3A	11	CD	M3 TG- GFD	GFD	5.4	2

These study subjects did not fit into the groups in Table 2 and were accounted for as single study subjects. However, they were grouped into contexts. Study subjects who did not receive a celiac disease (CD) diagnosis at the time of the biopsy sampling for this study, but received a CD diagnosis at a later biopsy sampling (CD later), and study subjects who received a Not CD diagnosis at a later biopsy sampling (Not CD). Other subjects were included as control biopsies on a gluten-free diet (GFD) after a previous CD diagnosis; some of these subjects returned to a Marsh 0 histology (normalized CD) but one did not, although levels of IgA autoantibodies against tissue transglutaminase (anti-TG2) normalized (M3 TG- GFD). Other subjects had Marsh 3 histopathologies on a gluten-containing diet (GD) although their anti-TG2 levels were below the cutoff (M3 TG-). All of the study subjects were projected onto the principal component analysis in Fig. 2. Varying histopathologic assessments between pathologists are indicated by ranges in the Marsh grade column

^a^Levels of anti-TG2 analyzed in serum (study subject 7 analyzed in plasma)

^b^Number of HLA-DQ2.5*cis*. N/A = not available

^c^Study subjects 1, 2, 3, and 4 received their CD diagnosis at a biopsy sampling occasion 3, 10, 4 months, and 1 year and 7 months, respectively, after the biopsy sampling for this study

^d^Study subject 5 was judged not to have CD, after repeated sampling over a period of 4 years, based on normal histology and normalized anti-TG2 on GD

### Samples

Serum was sampled for diagnostic purposes, and levels of anti-TG2 and IgG antibodies against DG (anti-DG) were measured using EliA-kits from Thermo Fisher Scientific (Waltham, MA) and cutoff 7 U/mL according to Bragde et al. [23].

Biopsy specimens were collected using an endoscope (multiple specimens) or a pediatric Watson capsule (single specimen) for research and diagnostic purposes. Biopsies obtained using a Watson capsule (one study subject) were split into two pieces. For all of the subjects in this study, routine diagnostic histopathologic assessments were performed and reported using the modified Marsh scale (0, 1, 2, 3A, 3B, or 3C) [2, 3], according to Bragde et al. [17]. Because some of the subjects were included in an earlier study [17], additional assessments were available for some of the biopsies. The assessments (*n* = 21) were performed by a single pathologist. All available assessments were in consensus for RNA sequencing study subjects (Table 1) and for the real-time PCR validation study subjects used as baseline (Table 2). Varying Marsh grades between assessments were accepted for the remaining real-time PCR study subjects (Table 3). Biopsies for research purposes were immersed in pre-chilled RNAlater RNA Stabilization Reagent (Qiagen, Hilden, Germany) and total RNA was isolated according to Bragde et al. [17]. RNA concentrations were determined using a Qubit 2.0 Fluorometer and a Qubit RNA BR Assay Kit (Thermo Fisher Scientific, Waltham, MA) according to the manufacturer’s instructions for RNA sequencing samples, and using Nanodrop ND-1000 (Thermo Fisher Scientific Inc.) for real-time PCR samples. RNA integrity was assessed using an Agilent 2100 Bioanalyzer with the Agilent RNA 6000 Nano Kit (Agilent Technologies, Santa Clara, CA) according to the manufacturer’s instructions.

DNA was extracted from EDTA-treated blood using the Biorobot EZ1 and EZ1 DNA Blood 350 μL kits according to the manufacturer’s instructions (Qiagen).

### HLA-DQ2.5

The single nucleotide polymorphism (SNP) rs2187668 identified possession of the HLA alpha chain DQA1*05 and beta chain DQB1*02 alleles (HLA-DQ2.5) in *cis* efficiently in a study by van Heel et al. [24] and was, therefore, used as a measurement of the number of HLA-DQ2.5*cis* for each subject in this study. The SNP genotype was determined using assay C__58662585_10 and TaqMan Genotyping Master Mix (Life Technologies, Carlsbad, CA) with 20 ng of DNA in a total volume of 10 µL on a 7900HT Fast real-time PCR System using the standard thermal profile as recommended by the manufacturer (Life Technologies).

### RNA sequencing

Libraries for RNA sequencing were prepared using TruSeq Stranded Total RNA with Ribo-Zero Human/Mouse/Rat (Illumina, San Diego, CA) according to the manufacturer’s protocols with modifications, including automation using an Agilent NGS workstation (Agilent Technologies) and purification steps described by Lundin et al. [25] and Borgström et al. [26]. The libraries were clustered on a cBot and sequenced in multiplexes of ten libraries per lane on a HiSeq 2500 (Illumina) according to the manufacturer’s instructions using a read length of 1 × 50 bp. Demultiplexing and conversion were performed using CASAVA version 1.8.2. Sanger/phred33/Illumina 1.8 + was used as the quality scale.

Sample size estimations were performed using the software package PROPER version 1.10.0 [27] in RStudio version 1.0.143 [28] based on a public dataset with a high level of biological variation [29] and a two-group scenario. Simulations (*n* = 100) were performed based on a 5% expected rate of DEGs among a total of 26,000 genes using edgeR to detect DEGs at a false discovery rate (FDR) [30] of 5%. With these settings and with 20 samples in each group, the overall power to detect DEGs at an FC of 2, which was the FC cutoff that we used to select DEGs for further analysis, was 0.77. Excluding genes with an average expression ≤ 10 counts resulted in a power of 0.96. At gene counts of ten reads per sample, the average sized transcript of 2.2 kb [31] has an average reads per kilobase per million mapped reads (RPKM) value of 0.45 when sequencing at a depth of 20 million reads per sample when estimating that approximately 50% of the reads map to exons annotated in RefSeq. Based on this and on results from Ramsköld et al. [32], we determined that analyzing genes with average expressions > 0.3 RPKM was reasonable.

### Validation of RNA sequencing results and identification of potential biomarkers

A total of 29 genes with highly significant differential expression in the RNA sequencing analysis (Table 4, FDR-adjusted *p* value < 0.000001 and FC > 4 or FC < − 4) were selected for further real-time PCR analysis (ABI7900HT Fast Real-Time PCR System, Life Technologies). The selections were based on Gene Ontology (GO) terms to capture different aspects of CD. Analysis was performed using pre-designed gene expression assays dried down in 96-well plates (Online Resource 1), the Fast protocol, and TaqMan Fast Universal PCR Master Mix without AmpErase UNG, and with 10 ng of cDNA, converted from RNA using the High-Capacity cDNA Reverse Transcription Kit with RNase Inhibitor, in a total volume of 10 µL as recommended by the manufacturer (Life Technologies). Two reference genes were included for normalization; one reference gene, *EIF2B1*, was selected based on an evaluation in association with a previous study [17] and the other reference gene, *ZFR*, was selected among genes with an average expression > 1 RPKM and low variation between samples [smallest 95% confidence interval in relation to trimmed mean (5%); this study]. Additionally, the reference genes were evaluated for the absence of differential expression between the RNA sequencing study groups and the corresponding validation groups using one-way analysis of variance (ANOVA). The auto-baseline algorithm in the ExpressionSuite software package (version 1.1, Life Technologies) was used to compensate for background noise for each amplification curve, and thresholds were adjusted to the log-linear range and set to the same level for all of the samples in one assay. The data were then normalized against the two reference genes using the Genex software package version 5.4.2.128 (MultiD Analyses, Göteborg, Sweden).

**Table 4:** Highly significantly differentially expressed genes (HDEGs) were identified by comparing RNA sequencing data from study subjects with active celiac disease (CD) (Marsh 3, group M3, Table 1) with study subjects without CD (Marsh grade 0, group M0, Table 1) using two different approaches, one-way analysis of variance (ANOVA) or modeling of mean–variance relationships of count data using a lognormal distribution with shrinkage and differential expression analysis using linear regression (gene specific analysis, GSA)

Gene symbol	Gene name	FC RNA sequencing	FDR-adjusted *p* value ANOVA^a^	FDR-adjusted *p* value GSA^b^	FC real-time PCR (FDR-adjusted *p* value)
*ABCC2*	ATP binding cassette subfamily C member 2	− 5.1	1.5E−12	9.7E−12	
*ABCG5*	ATP binding cassette subfamily G member 5	− 4.8	2.1E−11	9.0E−14	
ACE*	Angiotensin I converting enzyme	− 4.6		6.3E−09	− 4.5 (1.1E−06)
*AGMO*	Alkylglycerol monooxygenase	− 5.1	5.2E−10	2.2E−11	
*ALDOB*	Aldolase, fructose-bisphosphate B	− 4.1	2.6E−14	3.7E−17	
*APOA1*	Apolipoprotein A1	− 41	6.3E−09	3.0E−15	
*APOA4*	Apolipoprotein A4	− 5.5		1.5E−08	
APOB*	Apolipoprotein B	− 5.1	4.5E−12	1.8E−18	− 4.7 (4.7E−08)
*APOC2*	Apolipoprotein C2	− 5.2	1.8E−08	2.3E−14	
APOC3*	Apolipoprotein C3	− 9.9	3.6E−10	6.7E−14	− 5.8 (1.9E−06)
*APOH*	Apolipoprotein H	− 9.1	9.7E−08		
*AQP10*	Aquaporin 10	− 6.6	1.9E−09	8.3E−14	
ASAH2*	*N*-acylsphingosine amidohydrolase 2	− 12	2.0E−16	8.8E−20	− 6.1 (1.1E−07)
*ASPHD2*	Aspartate beta-hydroxylase domain containing 2	4.7	1.5E−08	2.9E−14	
*BATF2*	Basic leucine zipper ATF-like transcription factor 2	4.6	5.0E−07	1.0E−13	
*CAPN13*	Calpain 13	− 4.6	2.6E−14	4.7E−18	
CAPN8*	Calpain 8	5.3		3.1E−09	5.2 (1.9E−06)
CD36*	CD36 molecule	− 4.9	2.6E−14	1.2E−15	− 3.2 (3.7E−07)
*CD79A*	CD79a molecule	4.2		8.6E−07	
*CEACAM20*	Carcinoembryonic antigen-related cell adhesion molecule 20	− 6.7		1.1E−09	
*CLSTN2*	Calsyntenin 2	− 4.8		3.3E−11	
*COL6A5*	Collagen type VI alpha 5 chain	− 4.5		7.2E−08	
CXCL9*	C-X-C motif chemokine ligand 9	5.5	6.8E−07	5.5E−10	3.6 (3.1E−06)
CXCL10*	C-X-C motif chemokine ligand 10	7.7		3.8E−11	5.7 (1.8E−07)
CXCL11*	C-X-C motif chemokine ligand 11	32		2.9E−15	22 (3.5E−08)
*CXCR2P1*	C-X-C motif chemokine receptor 2 pseudogene 1	5.1		4.9E−08	
*CYP2B7P*	Cytochrome P450 family 2 subfamily B member 7, pseudogene	− 12	2.7E−09	5.8E−14	
*CYP2C9*	Cytochrome P450 family 2 subfamily C member 9	− 5.7	9.6E−15	1.2E−17	
*CYP3A4*	Cytochrome P450 family 3 subfamily A member 4	− 33	8.9E−13	1.2E−17	
*DFNA5*	DFNA5, deafness-associated tumor suppressor	− 4.0		4.6E−11	
*DGAT2*	Diacylglycerol *O*-acyltransferase 2	− 10		1.3E−13	
*DIRAS2*	DIRAS family GTPase 2	− 7.3	9.0E−14	2.0E−12	
*ENPEP*	glutamyl aminopeptidase	− 5.1		1.9E−10	
*ENPP3*	Ectonucleotide pyrophosphatase/phosphodiesterase 3	− 11	2.3E−10	2.1E−17	
*F13B*	Coagulation factor XIII B chain	− 5.6	2.7E−07		
*FAM184A*	Family with sequence similarity 184 member A	− 5.5	5.4E−10	2.1E−10	
FCGR3A*	Fc fragment of IgG receptor IIIa	5.4		9.7E−11	N/A^c^
*G6PC*	Glucose-6-phosphatase catalytic subunit	− 15	1.7E−09	5.6E−14	
GBP5*	Guanylate binding protein 5	4.9	6.2E−07	9.7E−12	4.0 (3.5E−08)
*GSTA2*	Glutathione S-transferase alpha 2	− 5.6	1.7E−11	1.5E−09	
*HK2*	Hexokinase 2	7.5		6.7E−13	
*HMGCS2*	3-Hydroxy-3-methylglutaryl-CoA synthase 2	− 9.1	4.7E−09	1.1E−08	
IFI27*	Interferon alpha inducible protein 27	4.6		2.4E−09	3.2 (2.5E−06)
IFNG*	Interferon gamma	29	8.9E−08		17 (3.5E−08)
*IL1RN*	Interleukin 1 receptor antagonist	4.6		3.9E−08	
*IL21R*	Interleukin 21 receptor	4.9		3.6E−08	
LCN2*	Lipocalin 2	8.1		7.1E−09	12 (5.4E−06)
*LCT*	Lactase	− 20	1.7E−09	4.0E−12	
*LOC100507537*	Uncharacterized LOC100507537	− 7.7	3.4E−08	7.9E−11	
LPL*	Lipoprotein lipase	100		8.5E−17	107 (3.5E−08)
LRAT*	Lecithin retinol acyltransferase	− 9.6	4.7E−11	4.5E−16	− 6.4 (3.7E−07)
*MEP1B*	Meprin A subunit beta	− 4.3	9.0E−14	6.5E−15	
*MME*	Membrane metalloendopeptidase	− 4.6	2.6E−14	1.5E−15	
MMP3*	Matrix metallopeptidase 3	16		3.4E−09	10 (3.1E−06)
MMP12*	Matrix metallopeptidase 12	14	1.0E−06	1.0E−11	9.3 (7.7E−08)
*MS4A10*	Membrane spanning 4-domains A10	− 11	7.9E−14	4.7E−11	
*NELL2*	Neural EGFL like 2	− 6.1	4.8E−12	8.0E−18	
*NLRC5*	NLR family CARD domain containing 5	4.5	1.1E−07	2.4E−10	
PCK1*	Phosphoenolpyruvate carboxykinase 1	− 11	2.1E−10	1.4E−15	− 7.2 (4.7E−08)
*PCSK9*	Proprotein convertase subtilisin/kexin type 9	4.9		1.5E−07	
*PITPNM3*	PITPNM family member 3	4.6		1.4E−07	
PIWIL2*	Piwi like RNA-mediated gene silencing 2	− 4.1	1.2E−08	2.4E−10	N/A^c^
*PKLR*	Pyruvate kinase L/R	− 4.4	1.7E−08	8.0E−10	
*PON3*	Paraoxonase 3	− 6.0	2.2E−07	3.4E−10	
*PRKG2*	Protein kinase, cGMP-dependent, type II	− 9.8	1.8E−07	1.2E−15	
*RGN*	Regucalcin	− 6.6	5.0E−11	7.0E−14	
S100A9*	S100 calcium binding protein A9	4.8		6.1E−07	4.5 (1.1E−07)
*S100G*	S100 calcium binding protein G	− 5.1		2.4E−08	
*SCN3B*	Sodium voltage-gated channel beta subunit 3	− 10		2.6E−11	
*SI*	Sucrase-isomaltase	− 4.3	1.9E−09	5.5E−14	
*SLC2A2*	Solute carrier family 2 member 2	− 4.0	2.1E−09	6.9E−12	
*SLC5A11*	Solute carrier family 5 member 11	− 8.4	3.3E−10		
*SLC6A4*	Solute carrier family 6 member 4	− 4.8	1.9E−10	3.7E−10	
SLC6A14*	Solute carrier family 6 member 14	21		1.5E−09	21 (3.5E−08)
*SLC22A4*	Solute carrier family 22 member 4	− 6.5	6.6E−10		
*SLC23A1*	Solute carrier family 23 member 1	− 8.8	3.6E−11	2.0E−12	
*SLC28A2*	Solute carrier family 28 member 2	− 4.2	7.2E−07	4.4E−07	
*SLC46A1*	Solute carrier family 46 member 1	− 4.5	5.4E−11	3.2E−11	
SOAT2*	Sterol *O*-acyltransferase 2	− 14	6.4E−10		− 6.4 (3.7E−07)
*SPINK4*	Serine peptidase inhibitor, Kazal type 4	4.5		2.4E−10	
*SULT2A1*	Sulfotransferase family 2A member 1	− 6.8	3.3E−09	4.6E−08	
TFF1*	Trefoil factor 1	11		7.6E−07	6.1 (1.5E−06)
*TM4SF4*	Transmembrane 4 L six family member 4	− 5.7	5.7E−08	5.9E−10	
TNFRSF9*	TNF receptor superfamily member 9	6.8		7.6E−13	4.1 (3.5E−08)
*TREH*	Trehalase	− 5.4	2.3E−09	1.8E−11	
*TRPM6*	Transient receptor potential cation channel subfamily M member 6	− 8.0	1.9E−14	8.5E−17	
*TTC36*	Tetratricopeptide repeat domain 36	− 5.8	5.1E−08		
UBD*	Ubiquitin D	17		3.7E−12	8.3 (5.3E−07)
*UGT1A3*	UDP glucuronosyltransferase family 1 member A3	− 16	2.7E−09		
UGT1A4*	UDP glucuronosyltransferase family 1 member A4	− 15	3.3E−07		− 5.3 (6.8E−06)
*UGT2B7*	UDP glucuronosyltransferase family 2 member B7	− 6.3	8.7E−10	2.2E−13	
*UNC93A*	unc-93 homolog A	− 12	1.7E−12	2.9E−17	
UPB1*	Beta-ureidopropionase 1	− 35	9.3E−09		− 33 (7.7E−08)
VNN1*	Vanin 1	− 4.9	1.5E−12	3.0E−15	− 3.2 (4.7E−08)

Fold changes (FC) were based on mean expression (M3 vs. M0), and the *p* values were adjusted for multiple testing using false discovery rate (FDR). Genes marked with an asterisk were selected as potential CD biomarkers and validated using real-time polymerase chain reaction (PCR). Marsh grade 3 (group Active CD, *n* = 26, Table 2) vs. Marsh grade 0 (group Not CD, *n* = 10, Table 2) FCs from real-time PCR follow-up analyses are included, together with FDR-adjusted *p* values from the Mann–Whitney *U* test of differential expressions between the two groups

^a^One-way ANOVA using Partek Genomics Suite version 6.6 (Partek Incorporated, St. Louis, MO)

^b^GSA using Partek Flow version 5.0.16.0523 (Partek Incorporated)

^c^N/A = not available. Expression of *PIWIL2* and *FCGR3A* was not detected in a majority of the study subjects using real-time PCR, thus these genes were excluded from further analyses based on real-time PCR data

Calculations of fold changes and comparisons between real-time PCR results and RNA sequencing results were based on normalized relative quantification values and RPKM values, respectively. For genes with higher expression in Active CD than in Not CD subjects, the fold changes were equal to the mean expression ratio (Active CD vs. Not CD). For genes with lower gene expression in Active CD than in Not CD subjects, the fold changes were equal to − 1/mean expression ratio (Active CD vs. Not CD). All of the other statistics on the real-time PCR data were based on normalized values.

### Statistical analysis

In Partek Flow version 5.0.16.0523 (Partek Incorporated, St. Louis, MO), RNA sequencing data were aligned to genome build hg19 using STAR 2.4.1d [33], and the transcripts were then quantified using Partek E/M, an algorithm similar to an expectation/maximization algorithm published by Xing et al. [34] except that Partek E/M quantifies isoform expression levels across the whole genome at the same time and normalizes by transcript length. Refseq transcripts release 71 [35] was used as an annotation source. In addition, in Partek Flow, the mean–variance relationships of count data were modeled using a lognormal distribution with shrinkage (“limma trend” [36]), and differential expression was analyzed using linear regression (gene specific analysis [GSA]).

Partek Genomics Suite (version 6.6, Partek Incorporated) was used to further analyze RNA sequencing data by principal component analysis (PCA; with correlation as a dispersion matrix), Spearman rank correlation, and ANOVA, and to identify overrepresented gene groups as described by GO terms, including GO terms in the ontologies biological process, molecular function, and cellular component, using the Fisher’s test. To define relatedness between GO terms, the EnrichmentMap plugin [37] for Cytoscape version 3.4.0 [38] was used to visualize and cluster GO terms according to the Jaccard coefficient (similarity cutoff = 0.44). The clusters were described by word clouds with a maximum of ten words using the Cytoscape plugin Wordcloud version 3.1.0 [39].

The Partek Pathway (Partek Incorporated) was used for Pathway ANOVA to identify pathway level differential gene expression between study subjects with and without CD. Pathways [Kyoto Encyclopedia of Genes and Genomes (KEGG)] with 2–500 genes (*n* = 298) were included in the analysis, and least square means for all of the detected genes in a pathway were compared between study groups M3 and M0 using ANOVA.

Statistica version 13 (Statsoft, Tulsa, OK) was used to analyze the real-time PCR validation data by PCA (with correlation as a dispersion matrix), product-moment correlation, the Mann–Whitney *U* test, and Spearman rank correlation. Statistica was also used for hierarchical clustering (Euclidean distances and unweighted pair-group average) and for the Pearson’s *χ*^2^ test of independence.

Analysis of disease–gene associations for the potential CD biomarkers identified was performed using the R packages DOSE version 3.4.0 [40] and clusterProfiler version 3.6.0 [41] in RStudio based on the DisGeNET version 5.0 database [42], including gene sets with 10–500 genes.

Unless otherwise specified, all of the *p* values were adjusted for multiple testing using FDR, and FDR-adjusted *p* values < 0.05 were considered significant.

For the selection of highly differentially expressed genes (HDEGs), more stringent criteria were used: an FDR-adjusted *p* value < 0.000001 and FC > 4 or FC < − 4, and a mean expression > 1 RPKM in one or both study groups (ANOVA, Partek Genomics Suite) or a total number of reads > 1000 (GSA, Partek Flow).

Genes with mean expressions ≤ 0.3 RPKM were excluded from all analyses.

## Results

RNA sequencing libraries were successfully prepared from all of the samples, and a mean of 19.4 million reads per sample (13.9–23.5 million reads) was obtained for study group M0 and a mean of 20.7 million reads per sample (12.6–27.8 million reads) was obtained for study group M3. From a total of 26,369 annotated genes, 13,594 genes had mean expressions > 0.3 RPKM in the RNA sequencing data and were included in the analyses.

### Unsupervised grouping

In a PCA based on all of the genes with a mean expression > 0.3 RPKM, three principal components (PCs) each accounted for more than 10% of the total variation (PC1 = 27%, PC2 = 22%, and PC3 = 11%; visualized in Online Resources 2 and 3). Sample coordinates along these three PCs were analyzed using one-way ANOVA with respect to categorical variables (gender and Marsh grade according to Table 1) and using Spearman rank correlation with respect to continuous variables (age at biopsy in months, anti-TG2, and anti-DG) to identify relationships between these variables and the PC coordinates. Marsh grade was found to be associated with the coordinates along all three PCs (PC1, PC2, and PC3: FDR-adjusted *p* values = 0.024, 0.0018, and 5.9E−07, respectively), but no significant associations were found between PCA coordinates and gender.

Significant correlations with anti-TG2 and anti-DG were found for PC2 and PC3, with Spearman’s correlation coefficients (*r*_s_) and FDR-adjusted *p* values: anti-TG2 PC2 *r*_s_ = − 0.46 and *p* = 0.0043, anti-TG2 PC3 *r*_s_ = − 0.70 and *p* = 1.8E−06; anti-DG PC2 *r*_s_ = − 0.55 and *p* = 0.00035, anti-DG PC3 *r*_s_ = − 0.74 and *p* = 1.7E−07. No significant correlations were found with age at biopsy.

### Differential gene expression based on histopathology

Significant DEGs with FC > 2 or FC < − 2 between study groups M0 and M3 (Table 1) were identified using one-way ANOVA (*n* = 1034). A PCA based on these DEGs identified one tight cluster of M0 specimens and one wider cluster of M3 specimens. However, one biopsy specimen from study group M0 clustered within the M3 study group (data not shown) and was, therefore, excluded. A new differential expression analysis without the specimen rendered 1177 DEGs (Online Resource 4). In a PCA based on the 1177 DEGs, the previously excluded M0 study subject still clustered within the M3 study group (data not shown).

In a previous study, we identified a gene expression profile for CD consisting of eight genes, *APOC3*, *CYP3A4*, *OCLN*, *MAD2L1*, *MKI67*, *CXCL11*, *IL17A*, and *CTLA4* [17]. Hierarchical clustering of the RNA sequencing data on these genes in this study clustered the biopsy specimens correctly (Fig. 2). The M0 study subject, who clustered with the M3 study group according to all DEGs, also clustered with the M3 study group in this analysis.

Sixty-five of 79 candidate genes from 42 non-HLA risk loci for CD described by Withoff et al. [12] were present at levels > 0.3 RPKM in our study. Eleven of these 65 candidate genes were among the DEGs with FC > 2 or FC < − 2; ten had higher expression levels (*CCR3*, *CIITA*, *CTLA4*, *FASLG*, *HCFC1*, *IRF4*, *NCF2*, *POU2AF1*, *PVT1*, and *RUNX3;* FC range = 2.1–3.8) and one had lower expression levels (*TREH;* FC = − 5.4) in study group M3 than in study group M0. The observed number of DEGs among the CD candidate genes was higher than expected by chance (*p* = 0.018, Pearson’s *χ*^2^ test of independence). By decreasing the FC cutoff to 1.5, an additional ten CD candidate genes were included among the DEGs (*CCR2*, *ITGA4*, *ICOS*, *PLEK*, *CD28*, *IRAK1*, *UBASH3A*, *TAGAP*, *PHTF1*, and *FBXO48*; data not shown).

### Pathway analysis

A total of 197 pathways with significant differential expression between study groups M0 and M3 were identified using Pathway ANOVA. Of these, 87 pathways showed an FC > 10 or an FC < − 10 (Online Resource 5). Among these, 54 pathways involved genes that generally expressed lower levels of RNA in study group M3 biopsies than in study group M0 biopsies. Most of these 54 pathways were related to metabolism (e.g., carbohydrate, lipid, amino acid, and drug metabolism) and transporters (e.g., protein, carbohydrate, vitamin, and fat digestion and absorption). Additionally, genes that were part of the peroxisome proliferator-activated receptor (PPAR) signaling pathway expressed lower levels of RNA in study group M3 than in M0. The remaining 33 pathways involved genes that generally expressed higher levels of RNA in study group M3 biopsies than in study group M0 biopsies, and the pathways with the lowest *p* values related to protein degradation (e.g., phagosome, proteasome) and infection (e.g., bacterial invasion of epithelial cells and *Salmonella* infection). Furthermore, pathways related to paracellular permeability (e.g., tight junction and adherens junction), and pathways related to immune response (e.g., NOD-like receptor signaling pathway and antigen processing and presentation) were represented. Additionally, several of the 33 pathways were related to autoimmune conditions (Type I diabetes mellitus, systemic lupus erythematosus, and autoimmune thyroid disease).

### Enrichment analysis

We found that DEGs were significantly overrepresented in a total of 1051 of 8181 gene groups annotated to different GO terms (Online Resource 6). One hundred and forty-two GO terms reached FDR-adjusted *p* values of < 0.00001, and 117 of these formed clusters containing two or more GO terms (Online Resources 6 and 7) and were described by word clouds (Online Resource 6). Word clouds from the top GO terms included innate immune system, neutrophil migration, and stress response. Both bacteria and virus were included in the word clouds. Additionally, words relating to transportation, response to wounding, cytokine production, cell motility and chemotaxis, metabolism and catabolism, and membrane, extracellular, and nuclear components were represented.

### Validation of potential celiac disease biomarkers

A total of 94 HDEGs were identified, and 29 of these genes were selected for validation using real-time PCR (Table 4). In an attempt to capture different aspects of CD, the 29 potential CD biomarkers were selected based on highly significant GO terms (FDR-adjusted *p* value < 0.00001). Two reference genes, *EIF2B1*and *ZFR*, with no detectable differences in expression between study groups M0 and M3 in the RNA sequencing data (Table 1) were included. The mRNA levels of the 29 genes were measured for 51 study subjects (Tables 2, 3). Using the selected assays, *PIWIL2* and *FCGR3A* expression was not detected in a majority of the study subjects, thus these genes were excluded from further analyses.

For one study subject (Marsh grade 3C) in the Active CD group, because the *APOC3* mRNA result could not be interpreted, the mean *APOC3* expression of all of the study subjects with Marsh grade 3C histopathology in the Active CD group was used for that study subject.

Four subjects in study group M0 and four subjects in study group M3 were selected from the exploratory RNA sequencing samples (Table 1) for validation by correlation using real-time PCR and were included in groups Not CD and Active CD (Table 2), respectively. For 26 of the 27 potential CD biomarkers, the RNA sequencing and real-time PCR results correlated well (range *r* = 0.89–1.00), whereas *IFI27* showed a lower correlation between datasets (*r* = 0.62). The selected reference genes were not significantly differentially expressed between groups Not CD and Active CD in the real-time PCR validation set (FDR-adjusted *p* values: *EIF2B1 p* = 0.39 and *ZFR p* = 0.31).

There was significant differential expression between Marsh grade 0 [group Not CD (*n* = 10), Table 2] and Marsh grade 3 [group Active CD (*n* = 26), Table 2] using the Mann–Whitney *U* test for all 27 potential CD biomarkers (FDR-adjusted *p* values, Table 4). The Spearman rank correlations of the real-time PCR results with Marsh grade [Not CD (*n* = 10) and group Active CD divided into Marsh grades 3A (*n* = 8), 3B (*n* = 8), and 3C (*n* = 10)] were significant for all 27 potential CD biomarkers (negative correlations: *r*_s_ range = − 0.61 to − 0.85 with FDR-adjusted *p* value range = 7.5E−05 to 1.9E−10; positive correlations: *r*_s_ range = 0.78–0.91 with FDR-adjusted *p* value range = 4.6E−08 to 5.7E−13).

#### Principal component analysis (PCA)

**Fig. 3:**
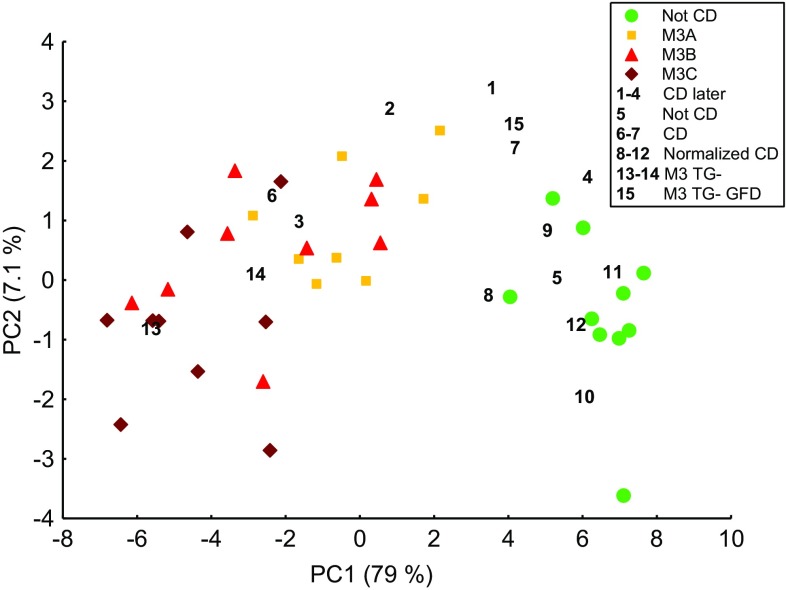
Coordinates of study subjects in a PCA based on the expression of 27 potential CD biomarkers (Table 4). Gene expressions of study subjects in groups Not CD and Active CD (Table 2) were used to construct the PCA and are represented in the PCA by colored markers. Study subjects 1–15 were projected onto this PCA and are represented by unique study subject numbers (Table 3)

A PCA was constructed using real-time PCR results from the 27 potential CD biomarkers for study subjects in groups Not CD and Active CD (Fig. 3, Table 2), thus forming a baseline. The remaining study subjects (1–15, Table 3) were not included in the PCA calculations because they were analyzed with the aim of exploring their gene expressions relative to the baseline. Instead, they were projected onto the PCA based on their expression of the 27 potential CD biomarkers (Fig. 3). All of the 27 potential CD biomarkers had comparable influences on the coordinates of the study subjects along PC1. Most of the biomarkers also had an influence on PC2 with the highest influences coming from *CXCL9* and *CXCL10*. The PCA showed a gradual progression from Marsh grade 0 to Marsh grades 3A, 3B, and 3C. Study subjects 8–12 (Normalized CD) were positioned within the group Not CD. The Mann–Whitney *U* test revealed no significant differences between group Not CD and Normalized CD subjects with regard to expression of the 27 potential CD biomarkers (FDR-adjusted *p* value range = 0.52–0.95).

M3 TG- subjects (13–14, Marsh 3C) clustered with the Active CD group, whereas study subject 15 on a GFD (M3 TG- GFD, Marsh 3A) was closer to the Not CD group. Both of the M3 TG- subjects were below 1 year of age, whereas the M3 TG- GFD subject was 11 years old. The anti-DG levels were 253 and 806 U/mL for the M3 TG- subjects and 6 U/mL for the M3 TG- GFD subject.

**Fig. 4:**
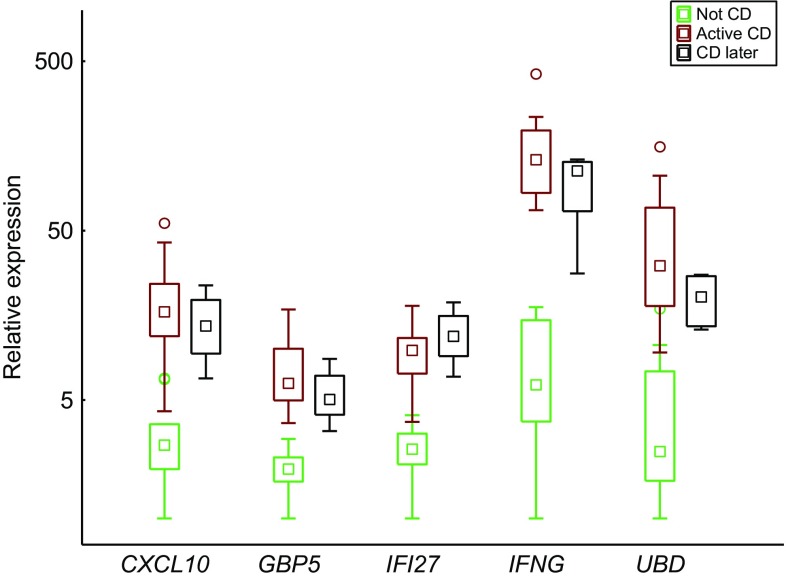
Box plot visualizing the expression of the five potential CD biomarkers that showed higher expression in subjects with no or low-grade intestinal injury who were later diagnosed with CD (CD later, Table 3) than in the Not CD group (Table 2) on a logarithmic scale. The box and the square within the box represent the 25–75% interquartile range and the median, respectively. The whiskers represent the non-outlier ranges

Study subjects 6 (Marsh grade 2–3B) and 7 (Marsh grade 2) received their diagnoses at the time of the biopsy sampling and projected near Marsh grade 3B study subjects and between Marsh grade 3A study subjects and the Not CD group, respectively. The CD later subjects (1–4) with no or low-grade intestinal injury were scattered from a position near Marsh grade 3B study subjects to a position near the Not CD group. Five genes, *GBP5*, *CXCL10*, *IFI27*, *IFNG*, and *UBD*, were significantly differentially expressed between the Not CD group and the CD later subjects (Mann–Whitney *U* test, FDR-adjusted *p* value range = 0.027–0.043), and there was a resemblance between CD later and the Active CD group (Fig. 4). Study subject 5, who had low-grade intestinal injury and was under investigation for CD at the time of the biopsy, but is no longer under investigation for CD, was positioned within the Not CD group (Fig. 3).

#### Associations with differential diagnoses

A total of 10,055 of 13,595 genes (all genes > 0.3 RPKM and *TNFRSF9*) were found in the DisGeNET database, which contained records of disease associations for 28 out of the 29 potential CD biomarkers. No association was found for *CAPN8*. A total of 484 significant disease associations were found, which involved combinations of 2–13 of the 28 biomarker genes represented in the database, including significant associations between CD and *APOB*, *IFNG*, *MMP3*, *S100A9*, *UBD*, *TFF1*, *TNFRSF9*, and *FCGR3A* of the biomarker genes (Online Resource 8). Focusing on inflammation/infection in the gastrointestinal tract, 17 disease–gene associations could be considered relevant, including e.g., inflammation, chronic ulcerative colitis, duodenal ulcer, enterovirus infections, and chronic gastritis (Online Resource 8). Based on this, 17 genes could be considered nonspecific for CD. The remaining twelve genes included *ASAH2*, *CAPN8*, *GBP5*, *LRAT, MMP12*, *PCK1*, *PIWIL2*, *SLC6A14*, *SOAT2*, *UBD, UGT1A4*, and *UPB1*.

### GO term clusters and potential biomarkers

Comparing the 29 potential CD biomarkers with the GO term clusters (all GO term clusters are found in Online Resources 6 and 7), clusters 1, 3, 5, 6, 8, 11, 16, and 18 included GO terms related to innate and adaptive immunity. The genes most frequently associated with these GO term clusters included *APOB*, *CD36*, *CXCL9*, *CXCL10*, *CXCL11*, *GBP5*, *IFNG*, *LCN2*, and *S100A9*. Clusters 2 and 7 included GO terms that related to components of the membrane and extracellular structures, and the genes most frequently associated with these GO term clusters included *ACE*, *APOB*, *APOC3*, *CD36*, *FCGR3A*, *LPL*, *PCK1*, *SLC6A14*, *UPB1*, and *VNN1*. Cluster 10 included GO terms relating to motility and migration, which included the genes *ACE*, *CXCL9*, *CXCL10*, *CXCL11*, *IFNG*, and *MMP3*. Clusters 2, 4, 17, and 19 included GO terms related to metabolic processes, and the genes most frequently associated with these GO term clusters included *ACE*, *APOB*, *APOC3*, *LPL*, *LRAT*, *UGT1A4*, and *UPB1*. Clusters 12 and 13 included GO terms related to transportation, which included the genes *ACE*, *APOC3*, *CD36*, *LCN2*, and *SLC6A14*. Clusters 9 and 14 included GO terms relating to nuclear nucleosomes, components of the chromosome, and DNA packaging, which included *PIWIL2*. Cluster 15 included GO terms related to negative regulation of viral genome replication and processes, but did not include any of the potential CD biomarkers.

## Discussion

In this study, we investigated gene expression in study subjects with CD and in non-CD study subjects by RNA sequencing of small intestinal biopsies to identify CD biomarkers and to investigate biological pathways involved in CD. Potential CD biomarkers were followed-up by real-time PCR in a separate group of study subjects with varying histopathologies and antibody levels. Previously, we created a gene expression panel to reflect crypt-villi architecture, the inflammatory response, and intestinal permeability to classify biopsies according to Marsh grade by screening a selection of potential biomarker genes [17]. In this study, we used an unbiased approach by selecting DEGs identified by RNA sequencing. Then, based on analyses of biological pathways, we selected a subset of the DEGs as potential CD biomarkers.

### Non-HLA risk loci genes

Based on RNA sequencing results, we identified 1177 DEGs. By comparing with CD candidate genes based on results from genome wide association studies [12], it was noted that out of 65 CD candidate genes (expressed at levels above the selected cutoff) eleven were included among the identified DEGs. Plaza-Izurieta et al. studied the expression of 45 CD candidate genes located in non-HLA CD risk loci [43]. Thirty-seven of those CD candidate genes were in common with those investigated in our study. Of the 37 genes, Plaza-Izurieta et al. identified 14 DEGs, whereas our study identified eight DEGs, and *CIITA*, *CTLA4*, *FASLG*, *PVT1*, and *TREH* were identified as DEGs in both studies. By reducing the FC requirement to 1.5, we identified five additional CD candidate genes (*CCR2*, *ICOS*, *PLEK*, *CD28*, and *UBASH3A*) which were also identified by Plaza-Izurieta et al. Discrepancies between the two studies may be attributed to differences in the gene sets analyzed, the genes considered as significantly differentially expressed, and to the different methodologies (fluidigm arrays vs. RNA sequencing) used. Increased expression of both *CIITA* and *CTLA4* has been associated with CD [17, 19, 44], and increased expression of the Fas ligand, which is encoded by *FASLG*, has been shown in lamina propria lymphocytes and intraepithelial lymphocytes in active CD when compared with non-CD controls [45].

Our analysis showed that candidate genes from CD-associated risk loci were overrepresented among genes that were differentially expressed between a histologically normal duodenal mucosa and a mucosa with typical CD lesions.

### Gene enrichment and pathway analysis

The 1177 DEGs were analyzed for overrepresentation in gene groups annotated to different GO terms, and additionally, pathways with differential mean gene expression in CD subjects compared with non-CD subjects were identified. These GO terms and pathways represented a number of different functions, many of which have been highlighted in other gene expression studies of CD biopsies using microarrays [14–16] and two dimensional difference gel electrophoresis [46] and in a microarray gene expression study of epithelial cells from individuals with active CD [18]. These studies are not directly comparable with each other or with this study due to differences in starting material, detection methods, and statistical methods for the analysis of biological context, but all of these studies still identified metabolism and cell cycle/proliferation. Other biological contexts shared with our study, although not shared with all of the studies, include immune response, cholesterol homeostasis, cell communication and organization, adhesion, transport facilitation, apoptosis, and antigen presentation. Pathways shared by this study and an RNA sequencing study of CD4 + T cells in CD [19] include pathways associated with metabolism and various autoimmune conditions.

Although one must be careful in the interpretation of results from pathway analyses based on gene expression in tissue samples made up of different cell types (e.g., small intestinal biopsies), we still wish to draw attention to some interesting findings, and to contextualize these findings in relation to current knowledge regarding CD. As such we have identified differential expression of genes involved in pathways associated with interactions with bacteria (e.g., bacterial invasion of epithelial cells, shigellosis, and *Salmonella* infection). Studies of the duodenal microbiota in children with active CD compared with non-CD controls have found an unbalanced microbiota associated with CD ([47–49], reviewed in [50]), and gene expression in epithelial cells from CD patients have indicated a possible response to CD-associated bacteria [44]. Increased intestinal permeability has been indicated in CD [51–53], and by systematic annotation of CD loci, Kumar et al. identified a subset of four CD-associated genes that are important in maintaining the function of the intestinal barrier [20]. Also our study indicate a disturbed epithelial barrier function with higher expression in active CD subjects compared with non-CD subjects of genes involved in for instance tight junction, adherens junction, and the regulation of actin cytoskeleton.

Our analysis showed that among pathways expressed at higher levels in CD lesioned duodenal mucosa as compared to non-CD mucosa were those relating to immune response, microbial infection, phagocytosis, and intestinal barrier function, while pathways relating to metabolism and transportation were expressed at lower levels.

### Potential biomarkers

Twenty-nine potential CD biomarkers were identified based on differential expression in small intestinal biopsies from CD and non-CD subjects and by information from highly significant GO terms. Both the combination of biomarkers as well as their expression profile may confer specificity for CD, but needs to be investigated. An analysis of disease-gene associations present in the DisGeNET database indicated that 17 of the potential CD biomarkers could be part of a general response to inflammation/infection in the gastrointestinal tract. Of the remaining 12 potential CD biomarkers, *ASAH2*, *CAPN8*, *GBP5*, *LRAT, MMP12*, *PCK1*, *PIWIL2*, *SLC6A14*, *SOAT2*, *UBD, UGT1A4*, and *UPB1*, seven are involved in metabolic processes: intracellular cholesterol esterification (*SOAT2* [54]), gluconeogenesis, glyceroneogenesis, and cataplerosis (*PCK1* [55]), esterification of retinols (*LRAT* [56]), metabolism of dietary sphingolipids (*ASAH2* [57]), amino acid transportation (*SLC6A14* [58]), glucuronidation of lipophilic substances (*UGT1A4* [59]), and synthesis of ß-alanine and ß-aminoisobutyric acid (*UPB1* [60]). Among the remaining biomarkers, *MMP12* is involved in degradation of the extracellular matrix [61]. *CAPN8* encodes a proteolytic enzyme and has been implicated in gastric mucosal defense in mice [62]. *PIWIL2* is associated with stem cell self-renewal, gametogenesis, and tumorigenesis [63]. *MMP12* [14, 61], *UBD* [14, 19, 64], *PIWIL2* [19], and *GBP5* [19] have previously been shown to be differentially expressed in CD subjects compared with non-CD subjects. In addition, relating to *UGT1A4*, UGT enzyme activity has been found to be lower in CD subjects than in non-CD subjects [65].

Our analysis did not reveal a general contribution to inflammation or infection in the gastrointestinal tract for almost half of the potential CD biomarkers.

### Gene expression in low-grade intestinal injury

Five of the selected biomarkers, *GBP5*, *CXCL10*, *IFI27*, *IFNG*, and *UBD*, showed higher expression levels in subjects with no or low-grade intestinal injury (Marsh grade 0–2) who later developed CD than in non-CD subjects, and the higher expression levels were comparable to expression levels in active CD subjects (Fig. 4).

In the enrichment analysis, *GBP5*, *CXCL10*, and *IFNG* were associated with clusters of GO terms related to immunity. Expression of the chemokine CXCL10 can be induced by IFN-γ, and *IFNG* and *CXCL10* expression has previously been shown to be higher in active CD than in non-CD controls [15–17, 66]. Intestinal *IFNG* expression has been shown to correlate with Marsh grade [67]. The protein encoded by *GBP5* belongs to a family of IFN-γ-induced p65 GTPases, is a marker of IFN-γ-induced classically activated macrophages, and is involved in NLRP3-mediated inflammasome assembly [68]. *UBD* is involved in the ubiquitin–proteasome system, participates in activation of the NF-κB pathway [64], and elevated expression of *UBD* has previously been linked to CD [14, 19, 64]. *IFI27* encodes a protein that is involved in apoptosis [69] and elevated expression of *IFI27* has previously been found in epithelial cells from CD patients when compared with controls [44].

Our analysis indicated the existence of differentially expressed genes in children who later received a CD diagnosis compared to those who did not, thus suggesting that an unbiased RNA sequencing analysis of this subgroup might shed light on important pathways in the pathogenesis of CD.

### Considerations

PC2 and PC3 in a PCA based on all detectable gene expressions correlated with anti-TG2 and anti-DG levels and with Marsh grade, but not with gender. However, these factors did not explain most of the variation in PC1, although Marsh grade accounted for some of the variation in PC1. Possibly the variation in PC1 could be explained by pathologic features not captured by Marsh grade. In addition, the cell compositions of the small intestinal biopsies (e.g., enterocytes, goblet cells, Paneth cells, and different immune cells) may be a factor because RNA expressions are means of expression in all of the various cell types when analyzing whole intestinal biopsies rather than specific cell types and this factor could also affect pathway and enrichment analyses.

For one of the RNA sequencing study subjects, the PCA analysis based on all of the DEGs resulted in a discrepant classification compared to the classification based on histopathology and serology. The same discrepant classification occurred using RNA sequencing data from the eight genes included in our previously suggested CD gene expression profile [17]. Although the levels of CD-specific antibodies were below cutoff in this subject, the level of antibodies to native gliadin was somewhat elevated (10 U/mL). The reason for the discrepancy between the histopathology and gene expression classifications is difficult to identify, but may result from a patchy distribution of intestinal lesions.

The objective of this study was to identify duodenal gene expression biomarkers for CD that can differentiate between patients who come to the clinic with suspected CD and are diagnosed with CD from patients who are not diagnosed with CD, as well as to follow mucosal recovery in patients on a GFD. Our data suggest that we have identified potential CD biomarkers that will accomplish this aim, however, we do not know if we can separate CD from differential diagnoses, such as duodenal Crohn disease or autoimmune enteropathy by gene expression profiling. Some of these potential biomarkers could also show the same patterns in differential diagnoses associated with inflammation or infection in the gastrointestinal tract as indicated by the disease–gene associations found for several of the genes. However, the level of increase or decrease in expression of some genes may differ between medical conditions, and the combination of genes that are expressed differentially may differ. Additionally, both the requirement of a response to GFD for a CD diagnosis and the incidence of differential diagnoses should be considered in this context. Nevertheless, the discriminatory capacity of the potential CD biomarkers cannot be fully estimated without the analysis of specimens from differential diagnoses. The presence of mild histological lesions (as in Marsh 1) in itself shows low specificity for CD and must be interpreted with caution [1]. In such cases, the result of a gene expression profile may be taken into account along with other factors, such as genetic background, symptoms, CD-specific antibodies, and response to a GFD. Presence of villous shortening and crypt hyperplasia in the small intestine has significantly higher specificity for CD, but these characteristics can occasionally be associated with differential diagnoses, such as autoimmune enteropathy, duodenal Crohn disease, or drug-induced mucosal injury [6]. A gene expression profile based on whole biopsies could be helpful in cases displaying villous shortening and crypt hyperplasia, for example, when suboptimal orientation of biopsy specimens hampers histopathologic assessment.

The analysis of gene expression in whole biopsies by real-time PCR is a rather straight-forward procedure, and the ability of biomarkers to diagnose CD and to follow mucosal recovery on a GFD was the main focus of this study. However, for future potential treatment strategies, the biological pathways involved in CD is of great interest, and hopefully this study can contribute also to that very important aspect of CD diagnostics.

## Conclusions

The results from this study indicated that in CD (1) there is differential expression of genes located in CD risk loci, (2) a large number of pathways are affected, involving amongst others immune response, microbial infection, phagocytosis, intestinal barrier function, metabolism, and transportation, (3) there is a potential to find gene expression CD biomarkers in duodenal mucosa, (4) differential expression is present already in low-grade intestinal injuries.

Gene expression should be investigated further, especially targeting low-grade intestinal injuries to find pathways and biomarkers involved in early stages of CD pathogenesis.

## Electronic supplementary material

Below is the link to the electronic supplementary material.

Online Resource 1 Pre-designed gene expression assays were used to detect expression of selected genes by real-time polymerase chain reaction (PCR) (XLSX 14 kb)

Online Resource 2 Plot of PC1 and PC2 from a PCA of all genes with an expression > 0.3 RPKM for the study subjects in Table 1 (PDF 5 kb)

Online Resource 3 Plot of PC2 and PC3 from a PCA of all genes with an expression > 0.3 RPKM for the study subjects in Table 1 (PDF 5 kb)

Online Resource 4 The 1177 significantly differentially expressed genes found when comparing RNA sequencing results from study subjects with active celiac disease (Marsh 3, group M3, Table 1) with RNA sequencing results from study subjects without a celiac disease diagnosis (Marsh grade 0, group M0, Table 1) using one-way analysis of variance in Partek Genomics Suite (Partek Incorporated, St. Louis, MO) (XLSX 116 kb)

Online Resource 5 The 87 pathways (database Kyoto Encyclopedia of Genes and Genomes; KEGG) with significantly different RNA levels in study subjects with active celiac disease (Marsh 3, group M3, Table 1) when compared with study subjects without a celiac disease diagnosis (Marsh grade 0, group M0, Table 1) (fold change > 10 or fold change < − 10) that were identified using Pathway ANOVA in Partek Pathway (Partek Incorporated, St. Louis, MO) (XLSX 16 kb)

Online Resource 6 Gene ontology (GO) terms identified by analyzing for overrepresentation of previously identified significantly differentially expressed genes between celiac disease (CD) and non-CD (Online Resource 4) in gene groups annotated to different GO terms using the Fisher’s exact test in Partek Genomics Suite (Partek Incorporated, St. Louis, MO). For each of the top 142 most significantly enriched GO terms, the potential celiac disease biomarkers included in the gene group were accounted for, and for 117 of the 142 GO terms, the cluster membership based on results from the EnrichmentMap plugin for Cytoscape version 3.4.0 was accounted for, along with the word clouds extracted using the Cytoscape plugin Wordcloud version 3.1.0 (XLSX 157 kb)

Online Resource 7 Enrichment map of 19 clusters (numbered 1-19) formed from 117 of the top 142 most significantly enriched GO terms (Online Resource 6) identified by analyzing for overrepresentation of previously identified significantly differentially expressed genes between CD and non-CD (Online Resource 4). Nodes represent gene sets with numbers indicating individual GO term IDs, and the thickness of the edges represents overlap between gene sets. The nodes are colored based on FDR-adjusted *p* values with the lowest *p* value (*p* = 1.6E−26, green) to the highest *p* value (*p* = 8.0E−06, turquoise) on a continuous color scale (PDF 477 kb)

Online Resource 8 Disease–gene associations for the 29 potential celiac disease biomarkers were identified using R packages DOSE version 3.4.0 and clusterProfiler version 3.6.0 in RStudio based on the database DisGeNET version 5.0, including gene sets with 10-500 genes. Gene ratio is the number of genes associated with the disease among the 28 of the 29 potential biomarkers that were present in the DisGeNET database. BgRatio is the number of genes present in the background gene set that has been associated with the disease. The 17 disease–gene associations we have considered relevant when focusing on inflammation/infection in the gastrointestinal tract are summarized in the top table (XLSX 51 kb)

## Abbreviations

CD: Celiac disease
Anti-TG2: IgA autoantibodies against tissue transglutaminase
HLA: Human leukocyte antigen
DEG: Differentially expressed gene
PCR: Polymerase chain reaction
GFD: Gluten-free diet
GD: Gluten-containing diet
DG: Deamidated gliadin
*r*: Product-moment correlation coefficient
FC: Fold change
Anti-DG: IgG antibodies against deamidated gliadin
SNP: Single nucleotide polymorphism
HLA-DQ2.5: HLA alpha chain DQA1*05 and beta chain DQB1*02 alleles
FDR: False discovery rate
RPKM: Reads per kilobase per million mapped reads
GO: Gene ontology
*EIF2B1*: Eukaryotic translation initiation factor 2B subunit alpha
*ZFR*: Zinc finger RNA binding protein
ANOVA: Analysis of variance
GSA: Gene specific analysis
PCA: Principal component analysis
KEGG: Kyoto encyclopedia of genes and genomes
HDEG: Highly differentially expressed gene
PC: Principal component
*r*_s_: Spearman’s correlation coefficient
*OCLN*: Occludin
*IL17A*: Interleukin 17A
PPAR: Peroxisome proliferator-activated receptor
NLRP3: Nucleotide-binding domain and leucine-rich repeat containing gene family, pyrin domain containing 3
