# Supplementary figures and images for: Celiac disease biomarkers identified by transcriptome analysis of small intestinal biopsies
Source: Cell Mol Life Sci. 2018 Aug 10;75(23):4385–401. doi: 10.1007/s00018-018-2898-5 (PMC6208765; doi:10.1007/s00018-018-2898-5)

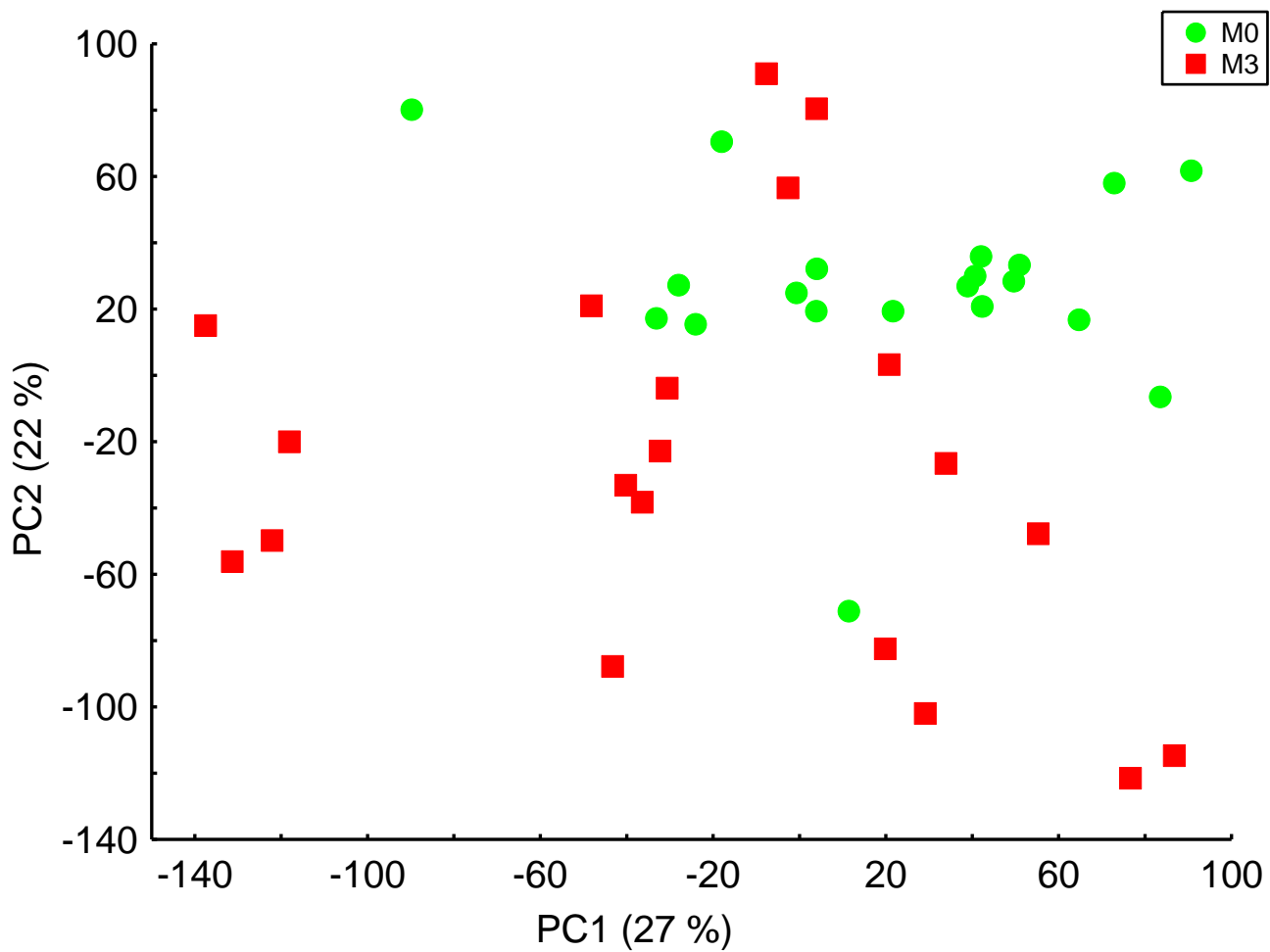

Supplement: Supplementary file 2 — Online Resource 2 Plot of PC1 and PC2 from a PCA of all genes with an expression > 0.3 RPKM for the study subjects in Table 1 (PDF 5 kb) [file 18_2018_2898_MOESM2_ESM.pdf]

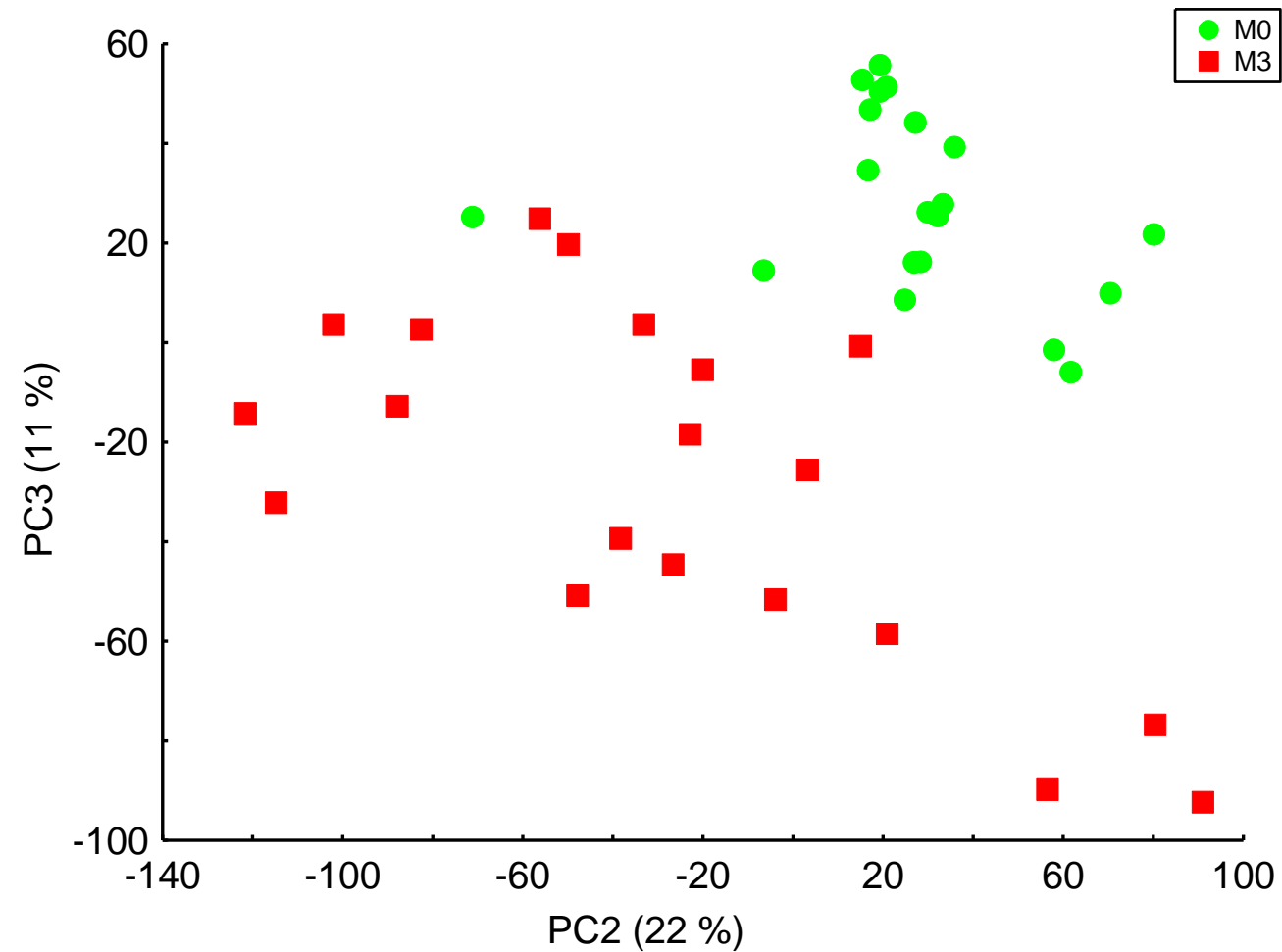

Supplement: Supplementary file 3 — Online Resource 3 Plot of PC2 and PC3 from a PCA of all genes with an expression > 0.3 RPKM for the study subjects in Table 1 (PDF 5 kb) [file 18_2018_2898_MOESM3_ESM.pdf]

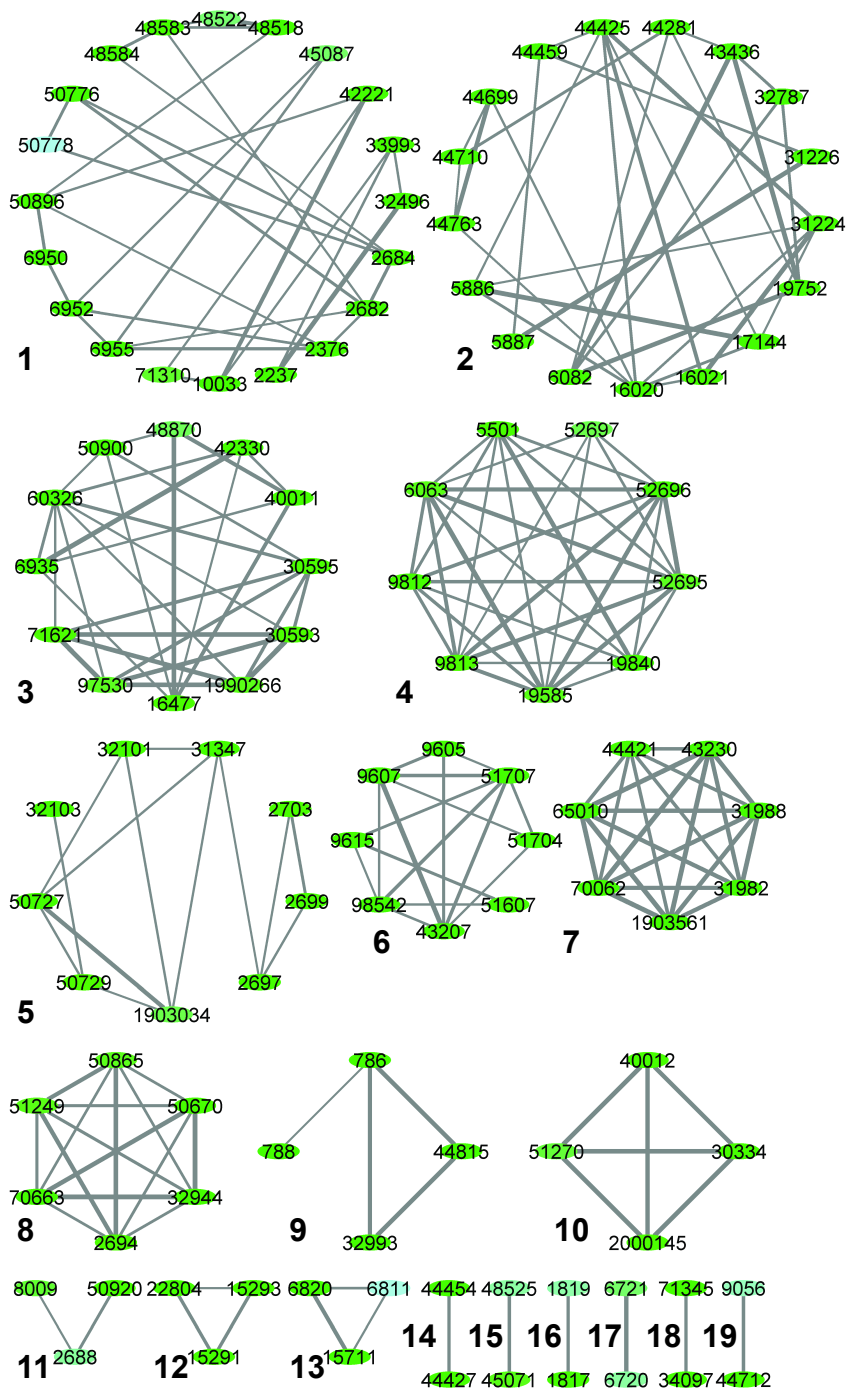

Supplement: Supplementary file 7 — Online Resource 7 Enrichment map of 19 clusters (numbered 1-19) formed from 117 of the top 142 most significantly enriched GO terms (Online Resource 6) identified by analyzing for overrepresentation of previously identified significantly differentially expressed genes between CD and non-CD (Online Resource 4). Nodes represent gene sets with numbers indicating individual GO term IDs, and the thickness of the edges represents overlap between gene sets. The nodes are colored based on FDR-adjusted p values with the lowest p value (p = 1.6E−26, green) to the highest p value (p = 8.0E−06, turquoise) on a continuous color scale (PDF 477 kb) [file 18_2018_2898_MOESM7_ESM.pdf]
